# Quality of life measures and health utility values among dry eye subgroups

**DOI:** 10.1186/s12955-018-0999-3

**Published:** 2018-08-31

**Authors:** Chika Shigeyasu, Masakazu Yamada, Motoko Kawashima, Kazuhisa Suwaki, Miki Uchino, Yoshimune Hiratsuka, Norihiko Yokoi, Kazuo Tsubota, for the DECS-J study group, Chika Shigeyasu, Masakazu Yamada, Motoko Kawashima, Kazuhisa Suwaki, Miki Uchino, Yoshimune Hiratsuka, Norihiko Yokoi, Kazuo Tsubota, Yoshitsugu Tagawa, Seika Den, Miki Iwasaki, Hiroshi Saito, Reiko Ishida, Aoi Komuro, Naoki Iwasaki, Harue Matsumoto, Tomoko Goto, Atsuko Kiyosawa

**Affiliations:** 1 Department of Ophthalmology, Kyorin University School of Medicine, Tokyo, Japan; 2 Department of Ophthalmology, Keio University School of Medicine, Tokyo, Japan; 3 Santen Pharmaceutical Co., Ltd, Osaka, Japan; 4 Department of Ophthalmology, Juntendo University Graduate School of Medicine, Tokyo, Japan; 5 Department of Ophthalmology, Kyoto Prefectural University of Medicine, Kyoto, Japan

**Keywords:** Cross-sectional study, Dry eye, Short tear film break-up time, Visual quality of life, Health utility assessment

## Abstract

**Background:**

To determine whether quality of life (QOL) and health utility are affected to the same extent among dry eye (DE) patients with short tear film break-up time dry eye (TBUT-DE) with minimal clinical signs were as severe as aqueous-deficient dry eye (ADDE).

**Methods:**

A multicenter cross-sectional study was conducted among DE patients who visited one of 10 eye clinics in Japan. Among the 463 registered patients, this study involved 449 patients with DE who were aged 20 years or older. Ophthalmic examination findings were assessed, including tear film break-up time (TBUT), Schirmer I value, and keratoconjunctival staining score. QOL was evaluated with the Dry Eye-Related Quality-of-Life Score (DEQS; 0 [best], 100 [worst]) and health utility (1 [total health], 0 [worst]) with the Health Utilities Index Mark 3 (HUI-3); scores were stratified by DE subgroup.

**Results:**

Median (interquartile range) of DEQS and HUI-3 scores across all participants were 21.7 (10.0–40.0) and 0.82 (0.69–0.91), respectively. Median (interquartile range) DEQS and HUI-3 scores in the ADDE group were 23.3 (10.0–40.0) and 0.79 (0.69–0.88), respectively; those in the short TBUT-DE group were 23.3 (13.3–38.3) and 0.82 (0.74–0.92), respectively. There were no significant between-group differences in questionnaire scores. Among the ophthalmic examination findings, a weak significant correlation between TBUT, corneal staining score and keratoconjunctival staining score to DEQS; TBUT and Schirmer test values to HUI-3, were seen.

**Conclusions:**

The burden of short TBUT-DE on QOL as assessed by the DEQS and HUI-3 was as severe as that in ADDE. Our findings suggest that clinicians should be aware of the impact of short TBUT-DE on patients QOL and utility values.

**Trial registration:**

University Hospital Medical Information Network (registration no. UMIN 000015890). Registered 10th December 2014, retrospectively registered.

## Background

Continuous efforts by researchers have produced substantial progress in the field of dry eye disease (DED). In 1995, the National Eye Institute/Industry Dry Eye Workshop defined dry eye (DE) as a disorder of the tear film due to tear deficiency or excessive evaporation that damages the interpalpebral ocular surface and is associated with symptoms and discomfort [1]. The International Dry Eye Workshop refined this definition in 2007, adding tear hyperosmolarity as a newly discovered cause of DE and addressing the instability of the tear film as well as the effect of DE on visual function [2]. National consensus definitions of DE for Japan were reported in 1995 [3] and 2007 [4].

Recently, a new definition of DE by the Asia Dry Eye Society has highlighted the important role of tear instability, based on evidence obtained from epidemiologic studies [5]. It has been reported that the majority of patients with DE have abnormal tear film break-up time (TBUT); in contrast, abnormal Schirmer tests and keratoconjunctival staining are less prevalent in this group [6, 7]. In addition to these objective DE tests, validated questionnaires including the Ocular Surface Disease Index [8], Impact of Dry Eye on Everyday Life [9], and Dry Eye-Related Quality-of-Life Score (DEQS) [10] questionnaires, have been developed for subjective evaluation of DE symptoms. The most common ocular symptoms of DED are discomfort and visual disturbance [2], including dryness, grittiness, ocular fatigue, redness, foreign body sensation, and soreness [6, 11]. These symptoms are known to reduce patients’ perception of their quality of life (QOL) [12, 13] and utility values [14, 15], which have identified a number of DE subtypes, including aqueous-deficient dry eye (ADDE) and short tear film break-up time dry eye (short TBUT-DE). Short TBUT-DE is characterized by severe symptoms with minimal ocular surface damage except for tear film instability [5, 7, 16]. The severity of the symptoms of short TBUT-DE is reportedly comparable with that of ADDE [17, 18]. In 2017, the epidemiological results of Dry Eye Cross-Sectional Study (DECS-J) revealed that the two most common DED subtypes were ADDE and short TBUT-DE [7]. These findings led us to speculate about whether the disease burden of DE subtypes (ADDE and short TBUT-DE) had a similar effect on QOL.

The importance of assessing the impact of disease on QOL from a patient-centric perspective has been established. Quantitative evaluation of patient-reported outcomes requires the use of measurement modalities with confirmed reliability and validity. The DEQS evaluates the severity of DE-associated symptoms and the multifaceted effect of DED on a patient’s daily life and has been evaluated for internal consistency, reproducibility, validity, and responsiveness when used in the Japanese population [10]. The Health Utilities Index Mark 3 (HUI-3) quantifies patient preferences for various health states and calculates associated health utility values [19, 20]. The HUI-3 is a standard method for assessing health utility. Schiffman et al. investigated health utility values using the time trade-off (TTO) method [14] and demonstrated that DE has a substantial impact on patients’ lives. However, there are no reports on the use of the HUI-3 to evaluate health utility values in patients with DED.

This study was performed to determine whether QOL and health utility are affected to the same extent in patients with short TBUT-DE with minimal clinical signs and those with ADDE. We compared the association of QOL and health utility among patients with ADDE and those with short TBUT-DE, using the DEQS and HUI-3. We also explored the associations of QOL and health utility with various ophthalmic parameters.

## Methods

### Ethics

The study protocol was approved by the Institutional Review Board of Clinical Study, Ryogoku Eye Clinic, Tokyo, Japan and registered in the University Hospital Medical Information Network registry (UMIN 000015890).

### Study subjects

The study subjects were previously included in the DECS-J [7]. That study collected data from 10 Japanese eye clinics that are broadly representative of the four main islands of Japan. To ensure the quality of the survey, two investigators’ meetings were held prior to the start of patient enrollment to discuss the study protocol and examination procedures.

The inclusion criteria were outpatients who were at least 20 years of age and were newly or previously diagnosed with DE were consecutively enrolled. The criteria for a diagnosis of DED were as follows: (1) at least one abnormal tear examination result (Schirmer I test ≤5 mm, TBUT ≤5 s); (2) abnormal results from ocular surface vital staining tests (fluorescein keratoconjunctival staining score ≥ 3), and (3) presence of DED symptoms [4]. Subjects who met two of the criteria (probable DE) or all three criteria (definite DE) were included in the study. The exclusion criteria were patients who have a history of hypersensitivity to fluorescein, patients with severe systemic disease, dementia, psychiatric illness, and severe functional impairment, such as paralysis or limb defects. Up to 50 patients were enrolled at each of the 10 study sites from December 1, 2014, to February 28, 2015.

### QOL and health utility assessment

QOL and health utility among patients with DE were evaluated using the DEQS questionnaire [10] and the HUI-3 [19], respectively. The DEQS is a functional questionnaire that assesses the subjective symptoms of DE; it consists of 15 questions and is scored using an overall summary scale and two multi-item subscales: bothersome ocular symptoms and impact on daily life. The bothersome ocular symptoms section includes 6 items: foreign body sensation, dry sensation in the eyes, painful or sore eyes, ocular fatigue, a sensation of heaviness in the eyelids, and red eyes. The impact on daily life section includes 9 items: difficulty opening the eyes, blurred vision, sensitivity to bright light, difficulty reading, watching television, or looking at a computer monitor or a cell phone display, feeling distracted because of eye symptoms, adverse effects of eye symptoms on work, staying at home because of eye symptoms, and feeling depressed because of eye symptoms. The score derived from this questionnaire is considered to be a quantitative measure of DED symptoms, in which 0 is the best score and 100 is the worst score. The test-retest reliability and discriminant validity of the DEQS were confirmed by a study in which the score in the DED group was significantly higher than that in the control group (33.7 vs 6.0) [10]. The DEQS is now widely used to assess the symptoms of DED [21, 22]. The HUI-3 consists of 15 questions that assess 8 health attributes: vision, hearing, speech, ambulation, dexterity, emotion, cognition, and pain. The HUI-3 scores were interpreted as health utility values using the method described by Furlong et al. [23]. A value representing the overall health state is derived by application of a weighted scoring algorithm in which 1 represents perfect health and 0 represents death, to provide a single index for clinical and economic evaluation of health care that is often used for cost-effectiveness analysis. The HUI has been confirmed to be a reliable and valid measure of health status [19]. Patients were asked to fill out the questionnaires at home and return them by post. The results were used to explore the association between subtypes of DE and ophthalmic examination values.

### Ophthalmic evaluation

The ophthalmic examinations included assessment of conjunctival and corneal vital staining with fluorescein sodium, measurement of TBUT, and the Schirmer I test. These examinations detect damage on the ocular surface and impaired tear function, and are used to diagnose DED.

Test strips containing fluorescein sodium (Fluores Ocular Examination Test Paper, Ayumi Pharmaceutical Co., Tokyo, Japan) were used for vital staining and TBUT measurement. Damage to the corneal and conjunctival epithelium was then evaluated by corneal fluorescein staining using the National Eye Institute grading system [1].

Corneal staining was graded with a score of 0 (minimum) to 3 (maximum) assigned to each of five corneal zones (superior, nasal, central, inferior, and temporal), with a maximum total score of 15. The fluorescein staining score of the keratoconjunctiva was determined according to the modified grading system of van Bijsterveld [24], wherein each eye is divided into three sections (temporal conjunctiva, cornea, and nasal conjunctiva) and scored from 0 to 3. The final score ranges from 0 (minimum) to 9 (maximum). The less staining score is interpreted as less damage to the cornea and conjunctiva. The Schirmer I test using test strips from Ayumi Pharmaceutical Co. was performed without topical anesthesia after all other examinations had been completed. To avoid any effect of keratoconjunctival staining on the Schirmer I test results, the tests were performed at least 15 min apart.

For each patient, the eyes that met the greatest number of criteria for a DED diagnosis was included in the study. If both eyes met the same number of criteria, the eye with (1) the higher fluorescein staining score and (2) the shorter TBUT was included; if these values were the same for both eyes, the right eye was used.

### Classification of DE subgroups

Subjects with DED were classified into a non-Sjögren ADDE subgroup and a short TBUT-DE subgroup based on ophthalmic examination findings [5]. The non-Sjögren ADDE group comprised subjects who fulfilled the following criteria: (1) presence of DE symptoms and (2) abnormal tear production (Schirmer I test value ≤5 mm). The short TBUT-DE subgroup included subjects who met the following conditions: (1) presence of DE symptoms, (2) abnormal tear stability (TBUT ≤5 s), (3) normal tear production (Schirmer I test value > 5 mm), and (4) no abnormality in the ocular surface vital staining test (keratoconjunctival score < 3).

### Statistical methods

SAS ver. 9.2 software (SAS Institute Inc., Cary, NC) was used for data analysis. Continuous data were expressed as median (interquartile range). The Mann–Whitney *U* test was used to assess differences between the two subgroups, and Pearson’s correlation coefficients were calculated to evaluate the strength of the associations between the two groups. Multiple linear regression analysis was conducted to adjust for age and sex. A *p*-value of < 0.05 was considered statistically significant. The HUI-3 results were calculated and interpreted as health utility values [23].

## Results

### Study populations

**Fig. 1:**
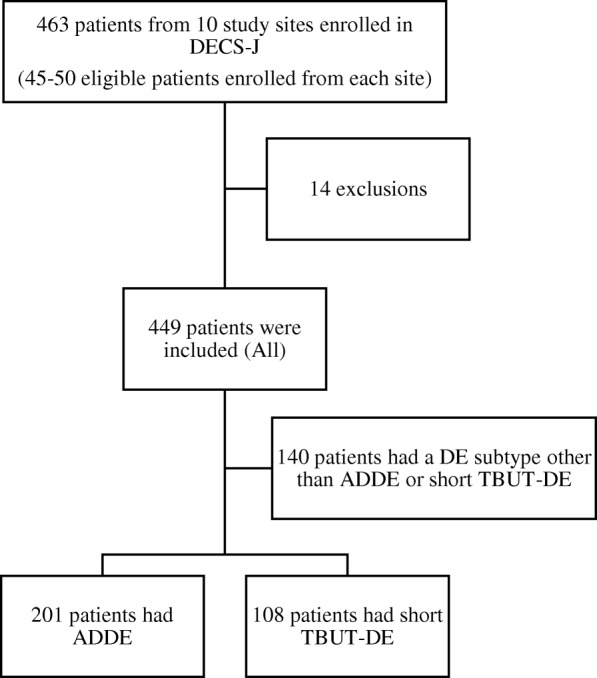
Study design. The population of the present study consisted of the two dry eye subgroups; the aqueous-deficient dry eye (ADDE, 201 patients) and short tear film break-up time dry eye (short TBUT-DE, 108 patients), from the 449 patients (All) included in the Dry Eye Cross-Sectional Study (DECS-J) [7]

A total of 463 eligible patients (45–50 per study site) were initially consented and 14 patients were excluded; 9 patients did not meet the criteria for diagnosis of DED, 3 patients were under 20 years of age and 2 patients drew consent. Four hundred and forty nine patients (63 men, 386 women; median age, 66.0 years) met the inclusion criteria and were included in the final analysis (Fig. 1). Response rates for the DEQS and HUI-3 were 99.1% (*n* = 445) and 96.9% (*n* = 435), respectively. Ophthalmic examination findings for TBUT were available for 99.6% of patients (*n* = 447), corneal and keratoconjunctival staining scores for 100% (*n* = 449), and Schirmer test results for 98.2% (*n* = 441).

### Characteristics of ADDE and short TBUT-type DED

The characteristics of all the DED patients included in the study (449 patients) and the two major DE subgroups; the ADDE (201 patients) and short TBUT-DE (108 patients) are shown in Table 1. The DE subtypes of 140 patients, those who were other than ADDE and short TBUT-DE include Sjögren syndrome, contact-lens related DE, meibomian gland dysfunction (MGD), friction-related DE, drug-induced DE, visual display terminal (VDT) related DE and others. Ophthalmic examination findings, including Schirmer test results, TBUT, keratoconjunctival staining scores, and corneal staining scores, were all significantly more severe in ADDE than in short TBUT-DE.

**Table 1:** Characteristics of the dry eye subtypes

	All^a^	ADDE	Short TBUT-DE
Number of patients (%)	449 (100%)	201 (44.8%)	108 (24.1%)
Age (y)	66.0 (52.0–75.0)	66.0 (55.0–75.0)*	63.5 (47.8–71.3)
Man: Woman	63: 386	30: 171	17: 91
TBUT	2.3 (1.7–3.7)	2.3 (1.7–8.3)*	3.0 (2.0–3.7)
Corneal staining score	3.0 (1.0–6.0)	3.0 (1.0–5.0)**	1.5 (0.0–4.0)
Keratoconjunctival staining score	3.0 (1.0–4.0)	3.0 (1.0–4.0)**	2.0 (1.0–2.3)
Schirmer test (mm)	6.0 (3.0–12.0)	4.0 (2.0–5.0)**	15.0 (9.0–25.0)

Abbreviations: *ADDE* aqueous-deficient dry eye, *TBUT* tear film break-up time, *TBUT-DE* tear film break-up time dry eye

Results are expressed as the median (interquartile range)

*p <  0.05, **p <  0.01 (Mann–Whitney U test) for ADDE subtype versus short TBUT-DE subtype

^a^“All” include 140 patients other than ADDE or short TBUT-DE subtypes, in addition to ADDE and short TBUT-DE subtypes

### QOL scores and health utility values

Table 2 shows the DEQS and HUI-3 values in patients with ADDE and those with short TBUT-DE. The median composite DEQS across all patients was 21.7 (10.0–40.0); scores for the two subscales were 29.2 (16.7–47.9) (bothersome ocular symptoms) and 13.9 (5.6–36.1) (impact on daily life). The mean HUI-3 score across all patients was 0.82 (0.69–0.91). The median composite DEQS scores in patients with ADDE and those with short TBUT-DE were 23.3 (10.0–40.0) and 23.3 (13.3–38.3), respectively (*p* = 0.93). Scores for the two subscales were also similar between ADDE and short TBUT-DE (bothersome ocular surface, 33.3 and 29.2, respectively, *p* = 0.75; impact on daily life, 13.9 and 16.7, respectively, *p* = 0.79). The median HUI-3 score in patients with ADDE was 0.79 (0.69–0.88), which was similar to that in patients with short TBUT-DE 0.82 (0.74–0.92) (*p* = 0.10). The results did not change in multiple linear regression analysis after adjustment for age and sex.

**Table 2:** Quality of life scores and health utility values among dry eye subtypes

	All^a^	ADDE	Short TBUT-DE
DEQS	21.7 (10.0–40.0)	23.3 (10.0–40.0)	23.3 (13.3–38.3)
Bothersome ocular symptoms	29.2 (16.7–47.9)	33.3 (16.7–50.0)	29.2 (20.8–45.8)
Impact on daily life	13.9 (5.6–36.1)	13.9 (5.6–36.1)	16.7 (5.6–33.3)
HUI-3	0.82 (0.69–0.91)	0.79 (0.69–0.88)	0.82 (0.74–0.92)

Abbreviations: *ADDE* aqueous-deficient dry eye, *DEQS* Dry Eye-Related Quality-of-Life Score, *HUI-3* Health Utilities Index Mark 3, *TBUT-DE* tear film break-up time dry eye

Results are expressed as the median (interquartile range)

^a^“All” include 140 patients other than ADDE or short TBUT-DE subtypes, in addition to ADDE and short TBUT-DE subtypes

### Correlation of ophthalmic examination values with QOL and utility values

Pearson’s correlation coefficients for the associations of DEQS and HUI-3 values with ophthalmic examination findings are shown in Table 3. Although the *p*-values of TBUT, corneal staining score and keratoconjunctival staining score showed significance to DEQS; TBUT and Schirmer test values showed significance to HUI-3, the correlation coefficients of TBUT, fluorescein staining scores, and Schirmer test values with DEQS and HUI-3 values were low.

**Table 3:** Correlation of ophthalmic examination findings with quality of life scores and health utility values

	DEQS	HUI-3
	*R* ^*2*^	*R* ^*2*^
TBUT	0.012*	0.182*
Corneal staining score	0.012*	0.008
Keratoconjunctival staining score	0.020*	0.002
Schirmer test	< 0.001	0.012*

Abbreviations: *DEQS* Dry Eye-Related Quality-of-Life Score, *HUI-3* Health Utilities Index Mark 3, TBUT, tear film break-up time

*p < 0.05, **p < 0.01 (Pearson’s correlation coefficient)

## Discussion

In this study, we evaluated the association of QOL with health utility values between the two major subtypes of DE, ADDE and short TBUT-DE. Short TBUT-DE, which is characterized by severe symptoms with minimal ocular surface damage except for tear film instability, is a new subtype of DE [11] that has gradually become more accepted. In the present study, we have shown that QOL and health utility were similarly severe in short TBUT-DE and ADDE, which features ocular surface damage accompanied by reduced tear volume. To the best of our knowledge, this is the first report to quantitatively evaluate the impact of DED on QOL and health utility using established questionaires, and to compare the results between patients with ADDE and those with short TBUT-DE.

In the present study, the median DEQS in patients with short TBUT-DE 23.3 (13.3–38.3) was comparable with that in patients with ADDE 23.3 (10.0–40.0). A significant correlation of DEQS scores with the results of the Short Form-8 Health Survey and the 25-item National Eye Institute Visual Functional Questionnaire has been reported [10]. Furthermore, DEQS scores have been reported to be significantly higher in patients with DED than in controls and to have improved significantly after treatment [10]. The authors of the presented research have previously found that of all the subtypes of DE, Sjögren syndrome was associated with the most severe score and MGD with the mildest score (38.8 and 18.1, respectively) [7]. The severity of the subjective symptoms of short TBUT-DE has been assessed using different QOL questionnaires. In one analysis of symptom scores in DE patients who visited the subspecialty outpatient clinic of a university hospital, Shimazaki-Den et al. found that the sum of the mean values of five symptom scores did not differ significantly between the ADDE and short TBUT-DE subgroups [17]. Another report by Yokoi et al. [18] used two questionnaires to assess subjective symptoms in VDT workers with either definite DE or short TBUT-DE [8, 25, 26] and concluded that the DE symptoms in short TBUT-DE were comparable with those of definite DE. Using a validated questionnaire specifically designed for DE, our results have confirmed the previous findings.

It has been reported that the method used to collect utility data affects the utility value [27]. However, in this study, the median HUI-3 value across all patients was 0.82 (0.69–0.91), which is comparable with the results obtained by Schiffman et al. (0.78 for moderate DE) [14] and Buchholz et al. (0.68 for moderate DE) [15] using the TTO method. We used the scores on the HUI-3 for evaluation of utility values because this instrument is validated in Japanese and is administered in questionnaire format, rather than requiring an interview like the TTO and standard gamble (SG) methods. The TTO method directly assesses how long a period of perfect health is equivalent to the given period with a current disease status [28] and the SG method is based on a paired comparison in which the subject chooses the strategy of perfect health with probability of death or an intermediate health state [29]. Interestingly, the median health utility values in patients with short TBUT-DE 0.82 (0.74–0.92) and those with ADDE 0.79 (0.69–0.88) were comparable.

The degree of correlation between symptoms and ophthalmic examination findings in these conditions remains uncertain. Our results demonstrate a statistically significant but weak correlation of TBUT, keratoconjunctival staining, and Schirmer test results with DEQS and HUI-3 scores, which is in agreement with several previous reports [6, 13, 30, 31]. However, Nichols et al. found no association between clinical tests for DE and the five major symptoms of DE [6], while Sullivan et al. concluded that there was no consistent relationship between clinical tests and the Ocular Surface Disease Index score [31]. Similarly, Mizuno et al. reported a discrepancy between ocular surface findings and QOL scores assessed using the Visual Functioning Questionnaire and the 8-item Short-Form Health Survey [13]. Bron et al. [30] reported that tear osmolarity was the best index for assessing subjective symptoms in patients with DE; in our study, although measurement of tear osmolarity would have been interesting, it would have been prohibitively difficult to provide the required device to all participating clinics. There could be several reasons for the discrepancy between signs and symptoms, including a need for new clinical tests for DED, failure to recognize the signs of the disease, and the relationship with hypersensitivity. Studies are underway to explore these possibilities further.

TBUT was the only index that showed a statistically significant correlation with both DEQS and HUI-3 in the present study. This finding may reflect the fact that a short TBUT is apparent in all types of DE and it is an essential index for diagnosing DED in clinical practice [5]. However, the extremely low correlation coefficients for these relationships suggest that further interpretation of the results would be difficult; given that the symptoms of DE are influenced by various factors, including psychological state, the impact of DE cannot be explained by “unstable tear film” alone [32].

Advances in research in the field of DE have revealed that the prevalence of short TBUT-DE is substantial, especially among VDT workers [16], and that the symptoms of this condition are similar to those of ADDE [17, 18]. Present study also seem to support the prevalence of short TBUT-DE were substantial, among all patients included in the study. Although the cause of short TBUT-DE is still unclear, tear film instability due to decreased wettability of the corneal and conjunctival epithelia has been proposed; indeed, decreased expression of MUC5AC and MUC16 mRNA has been reported in both ADDE and short TBUT-DE [17]. The authors of the presented research have previously reported a statistically significant correlation between TBUT and wheat germ agglutinin fluorescence intensity (a marker of ocular surface mucins) [33]. A decrease in mucins may be induced by underlying or subclinical inflammation [34–36] or by hyperosmolarity [37], which has been shown to activate nociceptors such as transient receptor potential melastatin subfamily member 8 (TRPM8), a cold thermoreceptor [38]. TRPM8 is known to regulate ocular surface wettability and also triggers DE symptoms [39]. In the present study, the severity of DE symptoms in patients with short TBUT-DE seems to support the revised definition of DE proposed by the Asia Dry Eye Society, which the new definition assigns the essential role for TBUT assessment for diagnosing DED [5].

The present study had several limitations. First, although the 10 clinics included in the study were widely distributed across Japan, the sample may not have been large enough to be representative sample of all Japanese eye clinics and patients. Second, although all the study investigators were DE specialists and followed the same guidelines, there may have been some variations in their evaluations. For example, since the scoring system of vital staining is qualitatively evaluated, it may have influenced the ophthalmic evaluations. Third, patients who had already been treated for DE were included in the study. Three hundred and seventy-seven patients (84%) had already received treatment for DE and 72 (16%) were newly diagnosed with DE and were yet to be treated. DEQS and HUI-3 scores were not significantly different between treated and untreated patients. Finally, the study questionnaires were answered by patients at home, so we cannot exclude the possibility of environmental effects on our findings, such as the order of administration settings. The extent to which mode of administration may have influenced the questionnaire results is unknown; however, it has been reported that mode of administration does not have a major effect on the response to questionnaires regarding health-related quality-of-life measures [40].

## Conclusions

We have shown that DE-related QOL assessed by the DEQS and health utility evaluated by the HUI-3 are similarly severe in patients with short TBUT-DE and those with ADDE. However, associations of QOL and health utility values with ophthalmic examination findings were generally weak. Our findings suggest that patients with short TBUT-DE with minimal clinical signs is an important DE subgroup with severe symptoms affecting QOL, and should be targeted for clinical intervention.

## Abbreviations

ADDE: Aqueous-deficient dry eye
DE: Dry eye
DECS-J: Dry Eye Cross-Sectional Study in Japan
DED: Dry eye disease
DEQS: Dry Eye–Related Quality-of-Life Score
HUI-3: Health Utilities Index Mark 3
MGD: Meibomian gland dysfunction
QOL: Quality of life
SF-8: Short Form-8 Health Survey
SG: Standard gamble
TBUT: Tear film break-up time
TBUT-DE: Tear film break-up time dry eye
TRPM8: Transient receptor potential melastatin subfamily member 8
TTO: Time trade-off
VDT: Visual display terminal
VFQ-25: 25-item National Eye Institute Visual Function Questionnaire
